# Quartz sand as a phantom for urinary stone dust

**DOI:** 10.1111/bju.16810

**Published:** 2025-05-30

**Authors:** Bingyuan Yang, Aditi Ray, James Zhang, Ben Turney

**Affiliations:** ^1^ University of Oxford Oxford UK; ^2^ Boston Scientific Corporation Marlborough MA USA

**Keywords:** Endourology, lithotripsy, stone dust, research methodology, renal calculi, stone‐free rate

There is no universal consensus on which fragments can safely be left behind following ureteroscopy (if any). There is growing interest in achieving a true zero‐fragment status in patients [1, 2] and this has led to the development of technologies such as suction ureteric access sheaths and direct in‐scope suction [3]. This research demands a high‐quality model for urinary stone dust. The most commonly used phantoms for urinary stones are gypsum‐based phantoms such as BegoStone [4], but gypsum is highly soluble in water even after casting, resulting in >40% mass being lost when small submillimetre particles are involved [5]. The properties of quartz sand are described here, and it is proposed as a suitable phantom for stone dust.

The following candidate dust phantom materials were considered based on availability across a wide range of small particle sizes from <100 μm to >1 mm: quartz, glass, BegoStone, polyethylene, polystyrene, and polypropylene. Their physical properties were considered first for authenticity (in comparison to common human urinary stone types) and next for experimental viability.

Quartz sand was investigated in more detail as a potential model. Pure graded quartz sand (Sika AG, Baar, Switzerland) was used at three grain sizes: 0.06–0.3, 0.3–0.8 and 0.7–1.2 mm. Samples of this quartz sand were separated by size using wet sieving techniques described by Keller et al. [6]. The resulting size fractions were <63, 63–125, 125–250, 250–500, 500–1000, and 1000–2000 μm.

The fluid dynamic properties of quartz sand were compared to size‐matched calcium oxalate stone dust (separated in the same way) in a sedimentation experiment. Approximately 100 mg of quartz particles of each size fraction were placed in 10‐cm long cuvettes filled with 0.9% saline solution. These were rapidly rotated 180° and the time taken for the first particle to sediment to the bottom of the cuvette was measured, to mirror the methodology described by Keller et al. [6], and repeated five times.

The physical properties investigated were density and solubility in water, as these directly impact the fluid dynamics and experimental viability of particles. These properties are shown in Table 1 for the most common human stone types (calcium oxalate monohydrate and dihydrate, struvite, uric acid, cystine, and the calcium phosphate‐based brushite and hydroxyapatite) and the proposed phantom materials [4, 7]. Other properties such as hardness, compressive strength and tensile strength are not considered as these phantoms are not expected to be used for lithotripsy experiments.

**Table 1:** Density and solubility of types of urinary stone and proposed stone dust phantom materials. Properties of the monohydrate and dihydrate forms of calcium oxalate are reported together.

Material	Density, g/cm^3^	Solubility, mg/100 g H_2_O
Calcium oxalate	1.84–2.08	0.61
Struvite	1.701	16.92
Uric acid	1.89	6
Cystine	1.677	11.2
Calcium phosphate	3.14	2
Brushite	2.32	8.52
Hydroxyapatite	3.05–3.16	0.44
BegoStone	1.563–1.995	260
Glass (vitreous silica)	2.4–2.8	0
Quartz (crystalline silica)	2.65	1.08
Polyethylene	0.88–0.96	0
Polystyrene	0.96–1.05	0
Polypropylene	0.855	0

Both the densities and solubilities of the different chemicals in urinary stones are heterogeneous. Density ranges from 1.7 to 3.2 g/cm^3^, while solubility ranges over several orders of magnitude from 0.44 to 16.9 mg/100 g H_2_O. This means that there cannot be a single phantom material that accurately represents the density of all stone types. BegoStone can model the density of the least dense stone types while silica‐based materials and aluminium model a higher density, similar to calcium phosphates. Plastics are much less dense than any stone type and often less dense than water, which is extremely problematic for modelling fluid dynamics and precludes their viability.

When experimental viability is considered, it is advantageous to minimise water solubility in order to maximise consistency and reproducibility. This is because the amount of mass lost in solution depends not just on the water solubility of the chemical, but also the volume of fluid, temperature, surface area (i.e., particle sizes), time spent in solution, and flow. For this reason, BegoStone was excluded as a potential model, as a 5 × 5 × 5 mm cube of BegoStone can (when dusted, or with sufficient time) be completely dissolved in just 100 mL water.

Therefore, based on the physical properties, glass and quartz were considered as viable candidate phantoms that modelled higher density stone dust and are both essentially insoluble in water. Quartz was selected as the candidate of choice as it is crystalline like the stone compounds being modelled (as opposed to glass, which is amorphous), and the particles are slightly irregular as opposed to perfectly spherical glass microbeads.

Sedimentation experiments with quartz sand compared to size‐matched calcium oxalate monohydrate (COM) stone dust were performed according to methodology described by Keller et al. [6]. Particles >63 μm of both types behave as expected with smaller particles taking exponentially longer to sediment. Fine stone dust <63 μm intermittently clumps together resulting in inconsistent results (also noted by Keller et al. [6]), while quartz particles <63 μm continue to sediment more slowly without any observable clumping.

In conclusion, quartz sand is a suitable phantom for stone dust. The density is similar to calcium phosphate stones, the low water solubility ensures that results are consistent, and sedimentation matches modelled behaviour. Unlike actual human stone, it is readily available commercially in a chemically pure form without the contaminants found in river sand.

Quartz sand is a standardised, reproducible and widely available model for stone dust that can be applied to a wide range of laboratory experiments. The range of available sizes from <63 μm to >1 mm enables the use of dust mixtures tailored for any usage scenario, from evacuation of fine dust by in‐scope suction to clearance of larger particles by suction sheaths.

Limitations include the higher density compared to common stone types, such as COM and struvite, and differences in particle shape. If greater authenticity is required then these data suggests that COM is the most suitable stone type, although large quantities of chemically pure stone are required to generate sufficient quantities of dust.

## Disclosure of Interests

This research received funding from Boston Scientific Corporation. Aditi Ray and James Zhang are employees of Boston Scientific Corporation. Ben Turney is a consultant to Boston Scientific Corporation.
